# Clinical adverse effects of sodium-glucose cotransporter 2 inhibitors: Protocol for a systematic review and meta-analysis

**DOI:** 10.1097/MD.0000000000011853

**Published:** 2018-08-10

**Authors:** Hao Li, Fang-Hong Shi, Shi-Ying Huang, Shun-Guo Zhang, Zhi-Chun Gu

**Affiliations:** a Department of Pharmacy, Shanghai Children's Medical Center, Shanghai Jiao Tong University School of Medicine; b Department of Pharmacy, Renji Hospital, School of Medicine, Shanghai Jiaotong University, Shanghai, P.R. China.

**Keywords:** adverse events, inhibitors, meta-analysis, placebo, sodium-glucose co-transporter 2, type 2 diabetes mellitus

## Abstract

**Background:**

Sodium-glucose cotransporter 2 (SGLT2) inhibitors are a novel class of oral antidiabetic drugs, which mainly increase urinary glucose excretion through reducing renal glucose reabsorption. There is still a concern about the overall safety profile of SGLT2 inhibitors. In this systematic review and meta-analysis, we will assess the clinical adverse effects of SGLT2 inhibitors in type 2 diabetes mellitus.

**Methods:**

This systemic review and meta-analysis described in this protocol will be conducted to follow the Preferred Reporting Items for Systematic Reviews and Meta-Analyses guideline. We will search Medline, EMbase, the Cochrane library and the ClinicalTrials.gov Website from 1946 to June 2018. Studies will be screened by title, abstract, and full text independently in duplicate. Double-blinded, placebo-controlled, and randomized controlled trials reporting safety data of SGLT2 inhibitors will be eligible for inclusion. Outcomes will include adverse events (AEs) varying degrees and AEs occurring in ≥3% patients or AEs aroused concerns by the Food and Drug Administration (FDA). The assessment of risk bias and data synthesis will be performed using STATA software (version12, Statacorp, College Station, TX). Outcomes will be reported by risk ratios for dichotomous outcomes and weighted mean difference (WMD) for continuous outcomes and their 95% confidence intervals. Subgroup, sensitivity, regression analyses will be performed to evaluate intertrial heterogeneity and bias of the results. *I*^*2*^ statistic will be used to evaluate heterogeneity among studies.

**Results:**

This systemic review and meta-analysis will evaluate AEs occurring in ≥3% patients or AEs aroused concerns by the FDA of SGLT2i as compared to placebo.

**Conclusion:**

Our study will provide a comprehensive picture of AEs of SGLT2 inhibitors.

## 1 Introduction

The substantial increase in the incidence of type 2 diabetes mellitus (T2DM) (from 8.8% in 1999 to 11.7% in 2014) boost T2DM as one of the leading cause of death or disability all over the world.^[1,2]^ Guidelines of the American Diabetes Association in 2018 recommend metformin as first-line pharmacological therapy for the management of T2DM.^[3]^ The rest of hypoglycemic agents are used as second-line treatment option in whom glycemia control is not achieved with metformin, or unable to tolerate metformin due to gastrointestinal side effects or contraindications, for instance, renal insufficiency.^[3,4]^ Sodium-glucose co-transporter 2 (SGLT2) inhibitors, a neotype oral antidiabetic drugs, lessening blood pressure by reducing renal glucose reabsorption, and causing urinary glucose excretion, provide a new second-line choice for treating T2DM at the same time.^[3,5,6]^ Results from the majority randomized controlled trials (RCTs) published in the last 3 years suggested a favorable overall safety profile of SGLT2 inhibitors. Concerns about SGLT2 inhibitors–related adverse events (AEs) are, however, still accompanied in particularly infection-related effects, osmotic diuresis, ketoacidosis, acute kidney injury, and so on.^[6–10]^ Furthermore, the Food and Drug Administration (FDA) has issued the warnings about occurrences of urosepsis, pyelonephritis, ketoacidosis, acute kidney injury, bone fracture or bone mineral density, and leg and foot amputations during postmarketing studies of SGLT2 inhibitors.^[11–14]^ Although SGLT2 inhibitors have been evaluated or currently being evaluated in large-scale, long-term randomized trials and real-world trials, there is still a lack of comprehensive evaluation of its safety issues. Otherwise, inadequate knowledge about comparative risks of SGLT2 inhibitors lead to its prudent use in the treatment of T2DM. In this study, we will conduct a systematic review and meta-analysis to present an overview of the clinical effects events of SGLT2 inhibitors in subjects with T2DM.

## 2 Methods

### 2.1 Registration

The reporting of the review will follow the principle of Preferred Reporting Items for Systematic Reviews and Meta-Analyses guidelines and conduct following a priori established protocol (PROSPERO: CRD42018090153).^[15]^ Ethical approval is not required because this is a literature-based study.

### 2.2 Criteria for considering studies for this review

This review will include double-blinded, placebo-controlled RCTs irrespective of language. Adult patients with T2DM will be included. The intervention comparisons will constitute SGLT2 inhibitors (dapagliflozin, canagliflozin, empagliflozin, tofogliflozin, luseogliflozin, ipragliflozin, and ertugliflozin) versus placebo. Cointerventions with other antidiabetic agents or comparing with other hypoglycemic agents will be excluded. The duration of these studies should be at least 12 weeks.

### 2.3 Types of outcome measures

This protocol proposes to assess the overall safety of SGLT2 inhibitors, mainly including AEs varying degrees and AEs occurring in ≥3% patients or AEs aroused concerns by the FDA, such as urinary tract infections, genital tract infections, hypoglycemia, ketoacidosis, acute kidney injury, bone fracture–related effects, total withdrawals, death, and so on.

### 2.4 Search methods for identification of studies

**Figure 1:**
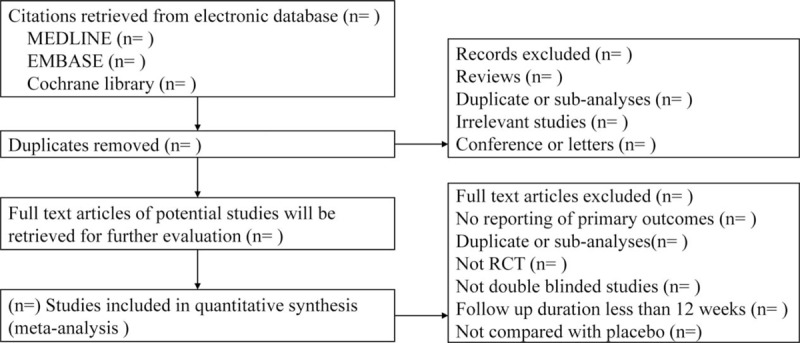
Flow chart of the search process. RCT = randomized controlled trial.

We will perform a comprehensive literature search of relevant databases including MEDLINE, EMBASE, Cochrane Library, and the ClinicalTrials.gov Website up to June 2018 to identify all double-blinded RCTs comparing SGLT2 inhibitors with placebo. Briefly, the search terms will include “sodium glucose cotransporter,” “SGLT2,” “SGLT-2,” individual names including trade name and their drug code of SGLT-2 inhibitors, “random” and “RCTs” (Table 1). Furthermore, we will search for additional eligible trials from the reference lists of other review articles, systematic reviews, and relevant publications. The details of the selection process are shown in Figure 1.

**Table 1:**
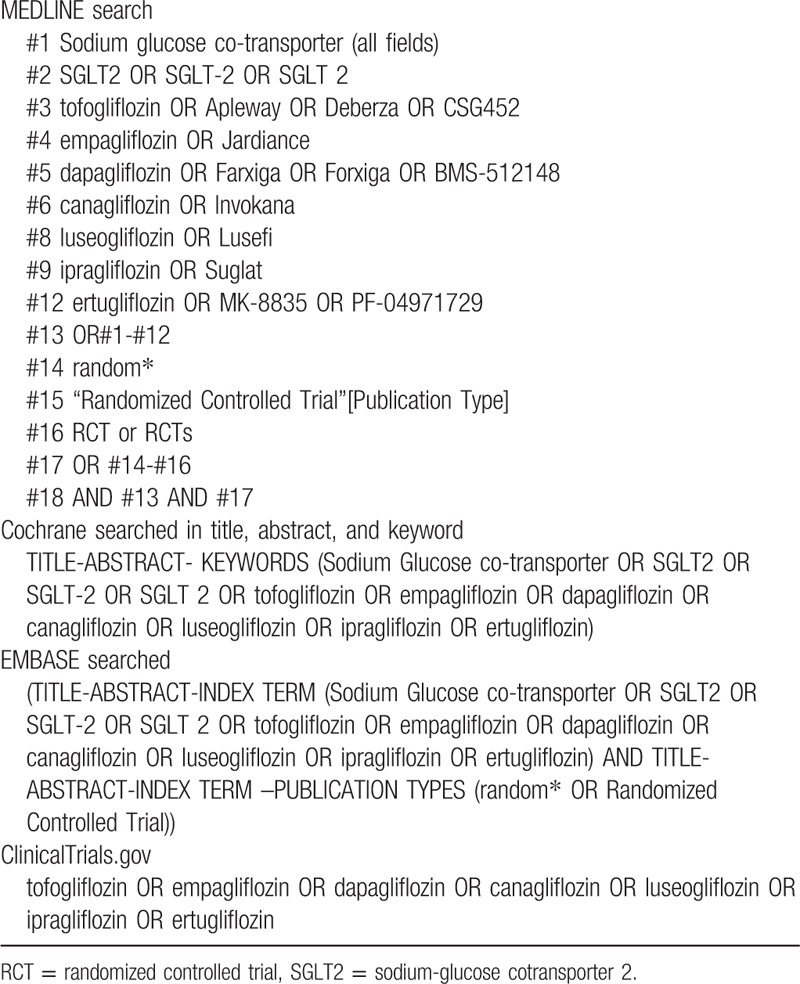
Electronic search strategies.

### 2.5 Data collection and analysis

Three authors (FHS, HL, and LS) will independently extract data and any disagreements are resolved by consensus or consulting a fourth author (ZCG). Literatures that are not conformed to the established inclusion criteria will be excluded. If the integrality of article is incomplete, we will email to the corresponding or first authors.

### 2.6 Data extraction

Electronic forms will be developed; the substantial contents of each selected articles will be extracted by 3 authors (FHS, HL, and LS), respectively; and the following data will be extracted: the name of the first author; NCT number; publication time; randomization; intervention characteristics such as type, dose, and duration of interventions; and patient characteristics such as background treatment, mean age, proportion of man, duration of type 2 diabetes, body mass index, glycosylated hemoglobin, and any safety data. Any disagreements are resolved by consensus or by consulting a fourth author (ZCG).

### 2.7 Assessment of risk of bias in included studies

We will use the *Cochrane Handbook for Systematic Reviews of Interventions* to assess bias risk, which include the following 7 items: randomization, allocation concealment, blinding (including participants and personnel and outcome assessment), incomplete outcome data, selective reporting, and other bias. Furthermore, we will judge these items by low risk, high risk, or unclear risk of bias. Any disagreement will be settled by discussion or consult to the fourth author.

### 2.8 Statistical analyses

Analyses will be performed by Stata V12.0 (STATA Corp, College Station, TX). Heterogeneity will be assessed by *I*^2^ values. *I*^2^ values <50% will be considered as acceptable heterogeneity, and >50% as considerable heterogeneity. When *I*^2^ values >50%, a random effects model with DerSimonian-Laird method will be used to assess the effect estimates, whereas *I*^2^ values <50%, a fixed effects model with Mantel-Haenszel method will be used. In addition, we will adopt qualitative description to analyze the data if quantitative synthesis is not appropriate.

### 2.9 Subgroup analysis

We plan to perform subgroup analyses based on different categories and dosages of each SGLT2 inhibitors.

### 2.10 Sensitivity analysis

We will conduct sensitivity analysis to identify the robustness of the result by omitting each of the study or excluding low-quality trials.

### 2.11 Reporting biases

We plan to perform funnel plots to identify potential reporting biases. In addition, we will also conduct Begg test and Egger test if asymmetry is showed by a visual inspection. There is no significant publication bias when *P* > .05 in Begg test and Egger test.

### 2.12 Ethics and dissemination

This systematic review does not require ethical assessment because only indirect literature will be included and evaluated. Furthermore, the result will be disseminated as a literature review in related journal.

## 3 Discussion

T2DM is a complex, progressive metabolic disorder characterized by persistent hyperglycemia and associated with macrovascular and microvascular complications.^[7]^ SGLT2 inhibitors can not only control glycemia but also reduce body weight and hypertension, which are particularly beneficial for T2DM accompanied by cardiovascular disease.^[16]^ SGLT2 inhibitors are generally considered as second-line treatment after metformin in T2DM. Previous studies have reported a great ability of glycemia control and cardiovascular benefits, but only few studies aroused concern of their adverse effects. Although some of the studies began to focus on the adverse effects of SGLT2 inhibitors such as infections, osmotic diuresis, ketoacidosis, acute kidney injury, and so on,^[6–10]^ there is still lack of studies to evaluate the overall adverse effects about SGLT2 inhibitors. Therefore, whether SGLT2 inhibitors can safely used in clinical is still need to elucidate. There is, however, still no previous relevant systematic review regarding the overall safety of SGLT2 inhibitors as compared with placebo.

The purpose of this review is to assess the overall clinical AEs of SGLT2 inhibitors in patients with T2DM. In particular, we will identify the influence of overall clinical AEs in different class and dosages of each SGLT2 inhibitors. Overall, we will give a comprehensive picture of SGLT2 inhibitors in safety. In order to ensure the accuracy and reliability of the results, different authors will screen articles at least 3 times, respectively. We intend to use sufficient evidence to guarantee credibility for this meta-analysis. Herein, this systematic review will be relatively comprehensive study to evaluate the safety of SGLT2 inhibitors in patients with T2DM. We hope that the results from this clinical systematic review will help inform the design of clinical trials.

### 3.1 Strengths and limitations of this study

- This is the first comprehensive review comparing the overall clinical adverse effects of 7 different SGLT2 inhibitors by meta-analysis.
- The results of this systematic review will help clinicians for decision making in clinical practice.
- The methods of this review are state of rigorous, including comprehensive literature search, explicit inclusion and exclusion criteria, independent study selection, data extraction, quality assessment, and statistical analysis.
- We limit our analyses to trials of SGLT2 inhibitors as compared with placebo, and double-blinded RCTs, which may be a limitation.

## Author contributions

HL, F-HS, and S-YH exacted and analyzed the data and wrote the first draft of the protocol, and F-HS helped with the design of the protocol and submitted the registration on PROSPERO. Z-CG and S-GZ provide professional support of this article, and Z-CG revised the manuscript. Z-CG is the guarantors for the publication and took the responsibility for the article. All authors participated in reading and approving the final manuscript.

**Conceptualization:** Hao Li, Fang-Hong Shi, Shi-Ying Huang, Shun-Guo Zhang, Zhi-Chun Gu.

**Data curation**: Hao Li, Shi-Ying Huang.

**Conceptualization**: Hao Li, Fang-Hong Shi, Zhi-Chun Gu.

## References

[1] Caspard H Jabbour S Hammar N Recent trends in the prevalence of type 2 diabetes and the association with abdominal obesity lead to growing health disparities in the USA: an analysis of the NHANES surveys from 1999 to 2014. Diabetes Obes Metab 2018;20:667–71. 29077244 10.1111/dom.13143 PMC5836923

[2] Lozano R Naghavi M Foreman K Global and regional mortality from 235 causes of death for 20 age groups in 1990 and 2010: a systematic analysis for the Global Burden of Disease Study 2010. Lancet 2012;380:2095–128. 23245604 10.1016/S0140-6736(12)61728-0 PMC10790329

[3] American Diabetes Association 8. Pharmacologic approaches to glycemic treatment: standards of medical care in diabetes-2018. Diabetes Care 2018;41:S73–85. 29222379 10.2337/dc18-S008

[4] Defronzo RA Banting lecture. From the triumvirate to the ominous octet: a new paradigm for the treatment of type 2 diabetes mellitus. Diabetes 2009;58:773–95. 19336687 10.2337/db09-9028 PMC2661582

[5] Musso G Gambino R Cassader M A novel approach to control hyperglycemia in type 2 diabetes: sodium glucose co-transport (SGLT) inhibitors: systematic review and meta-analysis of randomized trials. Ann Med 2012;44:375–93. 21495788 10.3109/07853890.2011.560181

[6] Puckrin R Saltiel MP Reynier P SGLT-2 inhibitors and the risk of infections: a systematic review and meta-analysis of randomized controlled trials. Acta Diabetol 2018;55:503–14. 29484489 10.1007/s00592-018-1116-0

[7] Li D Wang T Shen S Urinary tract and genital infections in patients with type 2 diabetes treated with sodium-glucose co-transporter 2 inhibitors: a meta-analysis of randomized controlled trials. Diabetes Obes Metab 2017;19:348–55. 27862830 10.1111/dom.12825

[8] Johnsson K Johnsson E Mansfield TA Osmotic diuresis with SGLT2 inhibition: analysis of events related to volume reduction in dapagliflozin clinical trials. Postgrad Med 2016;128:346–55. 26878357 10.1080/00325481.2016.1153941

[9] Erondu N Desai M Ways K Diabetic ketoacidosis and related events in the canagliflozin type 2 diabetes clinical program. Diabetes Care 2015;38:1680–6. 26203064 10.2337/dc15-1251 PMC4542268

[10] Tang H Li D Zhang J Sodium-glucose co-transporter-2 inhibitors and risk of adverse renal outcomes among patients with type 2 diabetes: a network and cumulative meta-analysis of randomized controlled trials. Diabetes Obes Metab 2017;19:1106–15. 28240446 10.1111/dom.12917

[11] Food and Drug Administration. 2015. FDA Drug Safety Communication: FDA revises label of diabetes drug canagliflozin (Invokana, Invokamet) to include updates on bone fracture risk and new information on decreased bone mineral density. Available at: https://www.fda.gov/Drugs/DrugSafety/ucm461449.htm. Accessed October 9, 2015.

[12] Food and Drug Administration. 2017. FDA Drug Safety Communication: FDA confirms increased risk of leg and foot amputations with the diabetes medicine canagliflozin (Invokana, Invokamet, Invokamet XR). Available at: https://www.fda.gov/Drugs/DrugSafety/ucm557507.htm. Accessed July 7, 2017.

[13] Food and Drug Administration. 2016. FDA drug safety communication: FDA strengthens kidney warnings for diabetes medicines canaglifozin (Invokana, Invokamet) and dapaglifozin (Farxiga, Xigduo XR). Available at: http://www.fda.gov/Drugs/DrugSafety/ucm505860.htm. Accessed August 6, 2016.

[14] Food and Drug Administration. 2015. FDA drug safety communication: FDA revises labels of SGLT2 inhibitors for diabetes to include warnings about too much acid in the blood and serious urinary tract infections. Available at: http://www.fda.gov/Drugs/DrugSafety/ucm475463.htm. Accessed January 12, 2016.

[15] Shamseer L Moher D Clarke M Preferred reporting items for systematic review and meta-analysis protocols (PRISMA-P) 2015: elaboration and explanation. BMJ 2015;350:g7647. 25555855 10.1136/bmj.g7647

[16] Zheng SL Roddick AJ Aghar-Jaffar R Association between use of sodium-glucose cotransporter 2 inhibitors, glucagon-like peptide 1 agonists, and dipeptidyl peptidase 4 inhibitors with all-cause mortality in patients with type 2 diabetes: a systematic review and meta-analysis. JAMA 2018;319:1580–91. 29677303 10.1001/jama.2018.3024 PMC5933330

